# BK virus infection, replication, and diseases in pediatric kidney transplantation

**DOI:** 10.1007/s00467-007-0462-x

**Published:** 2007-09-01

**Authors:** Philip D. Acott, Hans H. Hirsch

**Affiliations:** 1 Departments of Pediatrics and Pharmacology, Dalhousie University, Halifax, Nova Scotia, Canada; 2 Division of Pediatric Nephrology, IWK Health Center, 5850 University Avenue, Halifax, Nova Scotia, Canada; 3 Transplantation Virology, Medical Microbiology, University of Basel, Petersplatz 10, 4003 Basel, Switzerland; 4 Infectious Diseases and Hospital Epidemiology, University Hospital Basel, Basel, Switzerland

**Keywords:** Polyoma, BK virus, Children, Kidney transplantation, Immunosuppression

## Abstract

Polyomavirus-associated nephropathy is diagnosed in 2–8% of pediatric renal transplants and often precedes renal allograft dysfunction. Without intervention, however, significant graft dysfunction is observed in more than 50% of cases, although progressive early graft loss is reported in only three of 32 (9%) of cases. No specific treatment is available, but early decrease in immunosuppression is followed by declining human polyomavirus type 1 (BK virus) replication and improved outcome. The data suggest differences between pediatric and adult kidney transplantation. Possibly, pediatric patients might be able to mount a more vigorous BK virus-specific immune response than adult patients under similar modulation of immunosuppression. Also the role of cidofovir and leflunomide is still unresolved in pediatric patients. Larger prospective trials are needed to better define the impact of BK virus immunity for replication and disease as well as the role of reducing immunosuppression with or without cidofovir or leflunomide in pediatric transplant patients.

## Introduction

Kidney transplantation is the treatment of choice for children with end-stage renal disease, showing improving organ and patient survival over the past decade [1]. Part of this success is based on falling rates of acute allograft rejection, albeit at the expense of a rising number of infectious complications posttransplant [2]. In this setting, polyomavirus-associated nephropathy (PVAN) has emerged as one of the most formidable challenges [3–5]. PVAN has been initially described in adult patients [6, 7], and most data has been obtained from this patient population [8]. In fact, older recipient age of >50 years has been identified in some studies as an independent risk factor for PVAN [9]. However, PVAN also occurs in pediatric patients. For the purposes of this review, we will focus on human polyomavirus type 1 [BK virus (BKV)] infection, replication, and disease in pediatric kidney transplant recipients.

## BKV in the general population

Natural transmission of the BKV presumably occurs by respiratory or oral-enteric routes in children at a peak age of 2–5 years. Although no specific symptoms or signs have been identified, primary BKV replication may not necessarily be asymptomatic but in fact go unnoticed as a “flu-like” disease. Subsequently, BKV establishes nonreplicative, latent infection in renal tubular epithelial cells and uroepithelium [10]. The significance of detecting BKV in placental tissues or in cerebrospinal fluid of children with symptoms or signs of central nervous system disease requires further study [11]. Antibodies to BKV are commonly seen in 60–90% healthy adults, with most children being BKV seropositive by age 10 years [12, 13]. Differences have been observed between the conventional hemagglutination inhibition assay and the more sensitive enzyme immunoassays (EIA) techniques using BKV virus-like particles.

## Pediatric studies postrenal transplantation

Table 1 details relevant data regarding prevalence of BKV infection, replication, and disease in the pediatric renal transplant population. Alexander et al. [14] screened 52 pediatric renal transplant recipients for polyomavirus replication. BKV was detected in the urine of ten patients (19%), in plasma of seven (13.4%), and in allograft biopsies as PVAN in four (7.7%). Evaluation of BKV viruria by electron microscopy (EM) typically requires a viral concentration of 10^6^–10^7^ particles/ml and is therefore not as sensitive as polymerase chain reaction (PCR). This limited sensitivity may account for two cases of BKV viremia without concomitant viruria, or alternatively point to de novo infection in these patients. Ginevri et al. [15] conducted a prospective analysis of 100 pediatric renal transplant patents, with detectable BKV antibody in 70% at time of transplantation. By nested PCR assays, they found BKV viruria in 26%, BKV viremia in 5% of cases, three of whom progressed to BKV nephropathy during follow-up. In this study, mycophenolate mofetil (MMF) at baseline was associated with BKV nephropathy, whereas calcineurin inhibitor choice [tacrolimus vs. cyclosporin A (CyA)] and basiliximab induction were not associated with viruria, viremia, or nephropathy [15]. The role of immunosuppression for BKV replication was also demonstrated in a prospective case-control study of 18 Australian pediatric renal transplants with a seropositivity of 56% for BKV IgG, of whom 33% developed viruria as measured by PCR compared with no viruria in the age matched controls that had 39% seropositivity [16]. Herman et al. [17] found similar prevalence rates for BKV viruria (20%), BKV viremia (11%), and PVAN (4.3 %) in 46 patients followed prospectively. Of note, all of these children received antibody induction therapy in contrast to only 18% and 22% of patients studied by Ginevri et al. [15] and Haysom et al. [16], respectively (Table 2). Although more specific studies are needed, the data suggests that induction therapy does not influence prevalence of BKV replication and disease in the pediatric renal transplant population similar to reports in adult kidney transplantation [18], but differences between depleting and nondepleting antibodies and the small sample sizes in these studies should be acknowledged. The role of different calcineurin inhibitors cannot be answered conclusively, as no randomized controlled trials were performed. As can be gathered from Table 2, all combinations can be found among the cases of PVAN in children.

**Table 1:** Pediatric studies evaluating recipient human polyomavirus type 1 (BKV) antibody status, viruria, viremia, and/or nephropathy

Authors	Study methodology	Pt. number	Antibody detectable	Method (cutoff titre)	Viruria (BKV)	Method	Viremia (BKV)	Method	PVAN (BKV)
Alexander et al. [14]	Retrospective KT	52	N/A		19%	EM *	13%	PCR + RED	7.7%
Ginevri et al. [15]	Retrospective KT	100	70%	HIA (pos ≥ 1:40)	26%	Nested PCR	5%	Nested PCR	3%
Haysom et al. [16]	Prospective KT case control (age matched 1:1)	18	56% (39%)	IF IgG (pos ≥ 1:10)	33% (39%)	PCR + hybridization	6% (0%)	PCR + hybridization	0%
Herman et al. [17]	Prospective	46	N/A		20	Quantitative PCR	11%	Quantitative PCR	4.3%
Hymes et al. [36]	Prospective	122	N/A		N/A		16%	Quantitative PCR	6.6%
Muller et al. [37]	Cross-sectional KT, controls (KD, n = 35) (KDI, n = 7)	38	N/A		18% (KD 0%) (KDI 0%)	Nested PCR + RED	5% (KD 0%) (KDI 14%)	Nested PCR	3%
Smith et al. [26]	Retrospective, histology workup for PVAN	192	N/A (subgroup of PVAN)	BKV VLP (17%)	N/A	Quantitative PCR (100%)	N/A	Quantitative PCR (100%)	3.5%

*PVAN* polyomavirus-associated nephropathy, *N/A* not available, *EM* electron microscopy, *PCR* polymerase chain reaction, *RED* restriction enzyme digestion, *KT* kidney transplantation, *HIA* hemagglutination inhibition assay, *IF* indirect immunofluorescence (cell culture conditions not indicated), *KD* kidney disease, *KDI* kidney disease treated with immunosuppression, *VLP* virus-like particles, *Pt* patient, *requires > 10^6^ particles per ml and does not distinguish between BKV and JC virus

**Table 2:** Patient-specific data for pediatric studies evaluating recipient human polyomavirus type 1 (BKV) antibody status, viruria, viremia, and/or nephropathy

Authors	Induction therapy % antilymphocyte/% basiliximab	PRED (%)	CyA (%)	TAC (%)	RAPA (%)	MMF (%)	AZA (%)	DD/LRD (%)/(%)
Alexander et al. [14]	N/A	100	11	87	16	65	15	N/A
Ginevri et al. [15]	0/18	100	79	21	0	22	N/A	95/5
Haysom et al. [16]	0/22	100	61	39	0	56	N/A	83/17
Herman et al. [17]	100 (either)	100	54	41	N/A	28	63	78/22
Hymes et al. [36]	0/100	100	12.5	87.5	50	50	12.5	N/A
Muller et al. [37]	8/0	100	61	39	0	71	0	61/39

*PRED* prednisone, *CyA* cyclosporine, *TAC* tacrolimus, *RAPA* rapamycin, *MMF* mycophenolate mofetil, *AZA* azathioprine, *DD* deceased donor, *LRD* living related donor, *N/A* not available

Table 3 summarizes 32 cases of PVAN in pediatrics and the outcomes obtained in individuals younger than 20 years of age. The time point of first diagnosis of PVAN varies considerably from 1 to 48 months after transplant. It is noted that only three allografts (13%) were lost in this series, with the majority stabilizing with a reduction of immunosuppressive treatment. There was no uniform strategy for how to reduce immunosuppression as to drug switching, single drug reduction, and reduction of all immunosuppressants. As a common scheme, however, a 30% reduction in calcineurin-inhibitor dosing, a 50% reduction in antiproliferative drug dosing, and tapering of steroids to <10 mg has been common practice. Cidofovir was used in 16/32 patients (Table 3) and was associated with a favorable outcome in 13 cases (81%), which was not significantly different from that observed in patients treated solely with reduced immunosuppression of 11/15 (73%). The role of leflunomide cannot be evaluated from a single case with subsequent graft loss. The mechanism of action of leflunomide and cidofovir is under investigation (for review, see [19, 20]).

**Table 3:** Pediatric renal transplant patients with biopsy-proven human polyomavirus type 1 (BKV) nephropathy

Authors	Center BKV nephropathy rate (%)	Pt. number(<20 years)	Age (years)	Time posttransplant (months)	Treatment	Outcome; last creatinine
Alexander et al. [14]	7.7	4	N/A	38	↓ ImmunoSup	Stable graft function; Creat = 125 μM/l
			N/A	24	↓ ImmunoSup + cidofovir	Graft loss within 12 months
			N/A	12	↓ ImmunoSup + cidofovir	Deteriorating graft function; Creat = 240 μM/l
			N/A	6	↓ ImmunoSup	Stable graft function; Creat = 100 μM/l
Araya et al. [35]	N/A	3	8	48	↓ ImmunoSup + cidofovir	Graft function improved; Creat = 115 μM/l
			17	19	↓ ImmunoSup + cidofovir	Creat decreased from peak; Creat = 194 μM/l
			19	4	↓ ImmunoSup + cidofovir	Graft function near baseline; Creat = 88 μM/l
Comoli et al. [38]	N/A	3	9	32		Stable graft function; Creat = 106 μM/l
			15	3		Stable graft function; Creat = 123 μM/l
			18	1		Stable graft function; Creat = 132 μM/l
Ginevri et al. [15]	3	3	N/A	32	↓ ImmunoSup + cidofovir	Graft loss
			N/A	1	↓ ImmunoSup	Stable graft function; Creat = 132 μM/l
			N/A	4	↓ ImmunoSup	Stable graft function; Creat = 123 μM/l
Herman et al. [17]	4.3	2	13	6	↓ ImmunoSup + CMV Rx	Stable graft function; Creat = 114 μM/l
			8	14	↓ ImmunoSup + cidofovir	Stable graft function; Creat = 106 μM/l
Hymes et al. [36]	6.6	8	12 ± 4	22 ± 13	↓ ImmunoSup + cidofovir (7/8)	Stable graft function in 4/8
					↓ ImmunoSup (1/8)	Deteriorating graft function in 4/8; Creat = 150, 211, 97, 238 μM/l, respectively
Muller et al. [37]	3	1	N/A	N/A	↓ ImmunoSup + leflunomide	Graft loss
Smith et al. [26]	N/A	6	16	14	↓ ImmunoSup	Creat decreased from peak; Creat = 211 μM/l
			3	44	↓ ImmunoSup	Stable graft function; Creat = 115 μM/l
			8	47	↓ ImmunoSup	Deteriorating graft function; Creat = 282 μM/l
			5	4	↓ ImmunoSup	Stable graft function; Creat = 79 μM/l
			13	16	↓ ImmunoSup	Deteriorating graft function; Creat = 158 μM/l
			13	10	↓ ImmunoSup	Deteriorating graft function; Creat = 176 μM/l
Vats et al. [33]	N/A	2	4	22	↓ ImmunoSup + cidofovir	Creat decreased from peak; Creat = 150 μM/l
			10	12	↓ ImmunoSup + cidofovir	Creat decreased from peak; Creat = 158 μM/l

*N/A* not available, *CMV* cytomegalovirus, *Pt* patient, *ImmunoSup* Immunosuppression

Data from PVAN cases in adults suggest that steroids may be a risk factor for BKV replication and disease, but the role of reducing steroids as part of reducing immunosuppression is not clear. It is important to note that in most patients, BKV viremia will only start to decline for >1 log after 4–8 weeks. Breakthrough rejection was seen, and often, intense treatment of rejection preceded the recognition of PVAN, as has been reported in some adult studies [6, 18, 21].

Interstitial infiltrates at the time of diagnosis or following the reduction of immunosuppression in cases with PVAN are difficult to distinguish from acute rejection episodes. BKV-specific immune reconstitution has been associated with declining plasma BKV loads in the peripheral blood [22], which may follow the homing of BKV-specific lymphocytes to the sites of replication in the renal allograft. Considerable controversies exist over the role of adjunct diagnostic markers and the risk/benefit of short courses of steroids [8, 23]. Recent studies applying expression profiling [24] and urine proteomics [25] suggest new diagnostic tools, which are, however, faced with considerable data scatter, and will have to stand the test of prospective studies and the challenge of clinical practice.

Up to now, the data is not sufficient to determine whether or not primary BKV replication in seronegative kidney transplant recipients had a worse outcome compared with seropositive patients with secondary BKV replication. It can be speculated that patients with no or little BKV-specific memory compartment may need more time to mount sufficient control to terminate BKV replication. Only three children were identified as 5 years of age or younger. A retrospective study of 173 pediatric renal transplant patients had similar results, with six patients (3.5%) having PVAN a median of 15 months posttransplantation with functioning grafts during follow-up of a median of 28 months [26]. This study noted in the patients with PVAN that all had viruria (median = 6.1 million copies/ml), all had viremia (median = 21,000 copies/ml), and recipient seronegative status for BKV using the sensitive BKV virus-like particle enzyme-linked immunosorbent assay (ELISA) was a risk factor for developing PVAN [26].

**Fig. 1:**
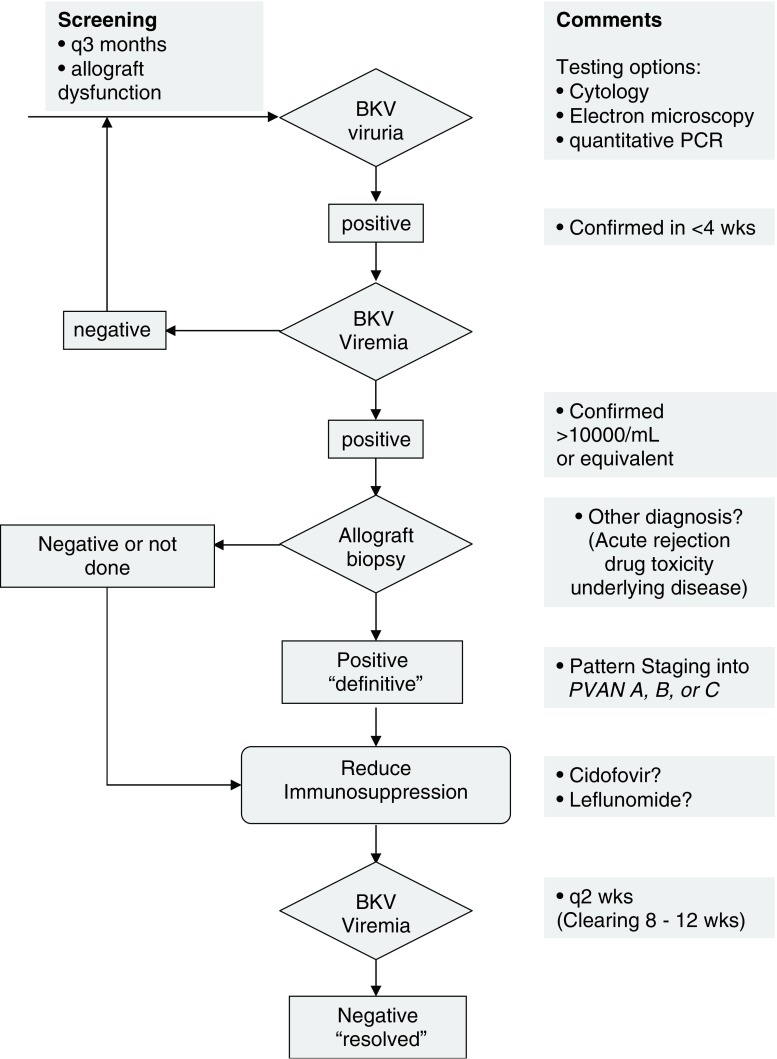
Screening and treating human polyomavirus type 1 (BKV) replication and disease in kidney transplant

BKV load in urine and blood has been found to be a helpful marker in identifying pediatric kidney transplant patients at risk of PVAN. Also, the course of plasma BKV loads has been used as a surrogate marker of allograft involvement, according to the results in adult patients [18, 27, 28]. At this point, however, no comparable pediatric data are available indicating that a plasma BKV load threshold of >10,000 copies/ml for >3 weeks is associated with a 93% sensitivity and specificity of histologically defined, e.g., definitive, PVAN. Even in adult patients, there are only few prospective studies addressing this issue. In a prospective study from the University of Maryland Transplant Center in Baltimore, MD, USA. Hirsch together with Drachenberg and Ramos reported a cut-off of 10,000 copies/ml was associated with a sensitivity of 68% and a specificity of 95% [29]. The lower sensitivity might be explained by a false negative biopsy result that, in retrospective studies, has been estimated to range from 10% to 30% [30] and might be higher in the prospective setting. Similar data for urine viral loads are lacking, and, despite the long-known relationship of urine viral loads being approximately 1,000-fold higher than plasma viral loads [31], no significant viruria threshold has been established. Thus, viruria currently serves as a very sensitive marker to exclude BKV replication but in the positive cases should be confirmed by viremia and eventually biopsy (Fig. 1).

## Oncogenic potential

The transforming potential of BKV infection has been noted early both in nonnatural experimental hosts and in in vitro tissue culture models. In these models BKV late gene expression/host cell lysis does not occur and the early gene mediated subversion of protooncogene and tumor-suppressor gene functions, particularly by large T-antigen function [4]. BKV-mediated oncogenicity is postulated in a report of a child with an adenocarcinoma of the donor renal allograft pelvis following PVAN virus allograft nephropathy, with regression of secondary tumors and a return to good health after removal of the primary tumor and cessation of immunosuppression [32]. Curiously, one of the pediatric BKVN patients reported by Vats et al. [33] presented with an echogenic mass that resolved with immunosuppression reduction and treatment with cidofovir. Rubio et al. [34] reported a child with PVAN and Epstein-Barr virus (EBV)-related posttransplant lymphoproliferative disease.

## Implications for screening programs in pediatrics

Although PVAN is generally thought to be the result of reactivation of BKV latent in renal tubules, it may represent a primary infection in younger children. This patient group may respond differently than patients with reactivation of latent virus from the transplanted kidney. Table 4 outlines pediatric patients who may be at higher risk of PVAN. These issues may become key considerations in children when developing a screening program, as both Ginevri et al. [15] and Smith et al. [26] noted recipient seronegativity for BKV antibody was significantly associated with risk of developing PVAN. In these pediatric patients, BKV viruria (six of six evaluated) and BKV viremia (five of five evaluated) preceded PVAN, as reported in adult patients by Hirsch et al. [18]. Alexander et al. [14] described two exceptions of viruria predating viremia, although this group used a less sensitive assessment of BKV viruria (EM for BKV) in comparison with PCR techniques. Herman et al. [17] did find correlation of BKV viremia with BKV viruria load and noted viruria predated viremia by a few weeks. However, as seropositive recipients may still develop BKV replication and disease, and more comprehensive data are lacking, it is currently not recommended to universally screen donors and pediatric transplant recipients for BKV serostatus but for BKV replication.

**Table 4:** Pediatric renal transplant risk factors for polyomavirus-associated nephropathy (PVAN)

Positive BKV viruria	BKV antibody mismatch	Recipient BKV antibody-negative status	Reduced BKV-specific cellular immunity
Decoy cells	D+/R–	R– and <5 years of age	Lack of BKV-specific interferon-γ-secreting lymphocytes in peripheral blood mononuclear cells
Electron microscopy			
Quantitative PCR			

*BKV* human polyomavirus type 1, *PCR* polymerase chain reaction, *D+* donor seropositive, *R–* recipient seronegative

## Summary and clinical relevance

PVAN affects 2–8% of pediatric renal transplants and often precedes renal allograft dysfunction. Significant graft dysfunction is observed in more than 50% of cases, although progressive early graft loss is reported in only three of 32 (9%) of cases. These estimates may reflect a reporting bias and are clearly limited by the lack of larger studies with sufficient follow-up. However, it cannot be excluded that pediatric patients might be able to mount a more vigorous BKV-specific immune response than adult patients under similar modes of immunosuppression. No specific treatment is available, although an improvement is often noted by a judicious decrease in immunosuppression. Dosing strategies to improve the efficacy and minimize the toxicity of cidofovir are emerging, as discussed by Araya et al. [35]. Clearly, randomized controlled trials are needed to prove any benefit of cidofovir in addition to reduced immunosuppression.

Understanding the role of BKV in transmission, target organ, risk factors, time frame of reactivation, and treatment options will be essential to improving transplant results in terms of patient morbidity, mortality, and graft survival. Understanding the contributions of viral infection and immune regulation in the pediatric renal transplant population would allow development of successful long-term strategies to minimize immunosuppressant drugs, viral injury, and rejection risk to pediatric patients with BKV infection. Until then, screening pediatric transplant patients for BKV replication by quantitative assays at least 3 monthly for the first 2 years and every time a biopsy is taken for cause or for surveillance will help to improve diagnosis and provide rationales for optimizing immunosuppressive treatment (Fig. 1).

## Abbreviations

AZA: azathioprine
BKV: BK virus
CAN: chronic allograft nephropathy
CMV: cytomegalovirus
Creat: creatinine (μM/liter)
CyA: cyclosporine A
DD: deceased donor
ELISA: enzyme-linked immunosorbent assay
EM: electron microscopy
HIA: hemagglutination inhibition assay
IF: indirect immunofluorescence
MMF: mycophenolate mofetil
LRD: living related donor
PCR: polymerase chain reaction
PVAN: polyomavirus-associated nephropathy
PRED: prednisone
RED: restriction enzyme digestion
SV40: simian virus 40
SRL: sirolimus
TAC: tacrolimus
